# Antibacterial activity of isopropoxy benzene guanidine against *Riemerella anatipestifer*

**DOI:** 10.3389/fphar.2024.1347250

**Published:** 2024-02-02

**Authors:** Yixing Lu, Weimei Qiao, Yaqian Xue, Xiaoxin Hong, Yuhang Jin, Jie Li, Xianfeng Peng, Dongping Zeng, Zhenling Zeng

**Affiliations:** ^1^ Guangdong Provincial Key Laboratory of Veterinary Pharmaceutics Development and Safety Evaluation, College of Veterinary Medicine, South China Agricultural University, Guangzhou, China; ^2^ National Risk Assessment Laboratory for Antimicrobial Resistance of Animal Original Bacteria, Guangzhou, China; ^3^ Guangzhou Insighter Biotechnology Co, Ltd., Guangzhou, China

**Keywords:** isopropoxy benzene guanidine, *Riemerilla anatipestifer*, antibacterial activity, membrane damage, resistance

## Abstract

**Introduction:** *Riemerella anatipestifer (R. anatipestifer)* is an important pathogen in waterfowl, leading to substantial economic losses. In recent years, there has been a notable escalation in the drug resistance rate of *R. anatipestifer.* Consequently, there is an imperative need to expedite the development of novel antibacterial medications to effectively manage the infection caused by *R. anatipestifer*.

**Methods:** This study investigated the *in vitro* and *in vivo* antibacterial activities of a novel substituted benzene guanidine analog, namely, isopropoxy benzene guanidine (IBG), against *R. anatipestifer* by using the microdilution method, time-killing curve, and a pericarditis model. The possible mechanisms of these activities were explored.

**Results and Discussion:** The minimal inhibitory concentration (MIC) range of IBG for *R. anatipestifer* was 0.5–2 μg/mL. Time-killing curves showed a concentration-dependent antibacterial effect. IBG alone or in combination with gentamicin significantly reduced the bacterial load of *R. anatipestifer* in the pericarditis model. Serial-passage mutagenicity assays showed a low probability for developing IBG resistance. Mechanistic studies suggested that IBG induced membrane damage by binding to phosphatidylglycerol and cardiolipin, leading to an imbalance in membrane potential and the transmembrane proton gradient, as well as the decreased of intracellular adenosine triphosphate. In summary, IBG is a potential antibacterial for controlling *R. anatipestifer* infections.

## 1 Introduction

*Riemerella anatipestifer* is a Gram-negative bacterium of the genus *Riemerella* in the family Flavobacteriaceae (Zhu et al., 2022). It incurs high morbidity and mortality rates among waterfowl, resulting in substantial economic losses for the poultry industry across various countries and regions (Tao et al., 2020). *R. anatipestifer* has numerous serotypes (Ke et al., 2022). Given that no cross immunoprotective effect occurs among these serotypes, vaccine development and disease control for *R. anatipestifer* infections remains challenging (Chu et al., 2015). Antibiotics are a rapid and effective means to treat the infection caused by *R. anatipestifer* (Tang et al., 2018). However, the widespread use and even abuse of antibiotics have led to the emergence and spread of clinically resistant *R. anatipestifer* strains (Nhung et al., 2017; Umar et al., 2021). Hence, it is of great significance to develop novel antimicrobial compounds for controlling infections caused by *R. anatipestifer*.

The guanidine group is one of the most important pharmacological groups in medicinal chemistry (Kapp et al., 2017; Gomes et al., 2023). Guanidine containing molecules are extensively used as anti-inflammatory, cardiovascular, antidiabetic and antihypertensive drugs (Song et al., 2019). Not least, many antimicrobial agents, such as the antibiotics streptomycin, trimethoprim and chlorhexidine or the antimalarial drug proguanil contain a guanidine group (Kim et al., 2021; Daily et al., 2022). These compounds are approved for clinical use in both human and animal medicine. Guanidine-containing compounds are often used as lead compounds in the research and development of various drugs (Saczewski eand Balewski, 2013). The guanidine functional group is positively charged and can bind to negatively charged bacterial cell walls or membranes through electrostatic interactions (Rauf et al., 2014). The insertion of other hydrophobic groups into bacterial cell walls or membranes causes cell membranes to rupture and induces bacterial death through cytoplasmic spillage (Wender et al., 2008).

**FIGURE 1:**
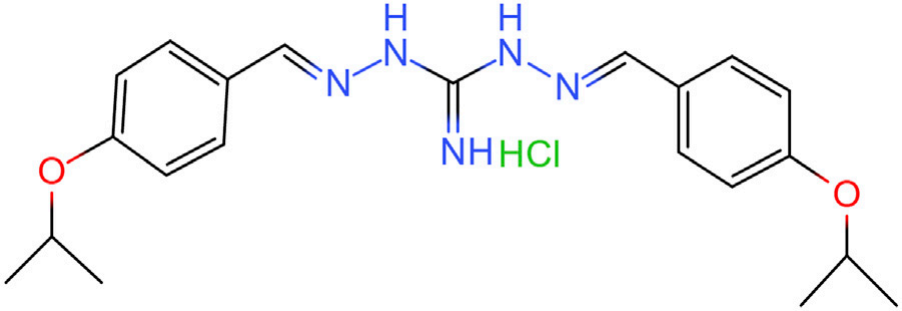
Chemical structure of IBG.

Isopropoxy benzene guanidine (IBG) is a guanidine derivative produced through the chemical condensation reaction of diaminoguanidine monohydrochloride with isopropoxy benzaldehyde. Its structural formula is shown in Figure 1. IBG has antibacterial activity against Gram-positive bacteria such as *Staphylococcus aureus*, *Clostridium perfringens*, and *Streptococcus suis* (Zhang et al., 2021; Lu et al., 2022; Han et al., 2023)*.* Although*,* IBG lacks antibacterial activity against some common Gram-negative bacteria such as *Escherichia coli* and *Salmonella*, it can restore the susceptibility of colistin-resistant bacteria when used in combination with colistin (Kong et al., 2022; Li et al., 2022). This compound exhibits favorable drug properties and holds potential as a leading compound in terms of its antibacterial activity and safety (Han et al., 2023). The objective of this study was to further investigate the antibacterial activity and mechanism of action of IBG against *R. anatipestifer*.

## 2 Materials and methods

### 2.1 Bacterial strains and chemicals

A total of 51 *R. anatipestifer* isolates were used. The isolates included ATCC11845 and 50 strains of *R. anatipestifer* isolated from duck farms (Supplementary Table S1). Tryptic soy broth (TSB; Huankai, Guangzhou, China) or tryptic soy agar (TSA; Huankai, Guangzhou, China) were used for the routine growth of *R. anatipestifer*. *R. anatipestifer* strains was inoculated overnight at 37°C in 2 mL of TSB with agitation at 180 rpm. IBG (99.9%) was synthesized by Guangzhou Insighter Biotechnology (Guangzhou, China). BCECF-AM was purchased from Shanghai Bioscience (Shanghai, China). DiSC_3_(5) was bought from Aladdin Industrial Corporation (Shanghai, China). Propidium iodide (PI) and enhanced adenosine triphosphate (ATP) assay kits were obtained from Beyotime Biotech Inc. (Shanghai, China). Gentamicin (GEN), ethylenediamine tetraacetic acid (EDTA), and Trixon-X-100 were acquired from Sangon Biotech (Shanghai, China). Phosphatidylglycerol (PG), phosphatidylethanolamine (PE), and cardiolipin (CA) were procured from Sangon Biotech (Shanghai, China).

### 2.2 Antimicrobial susceptibility testing

The minimal inhibitory concentrations (MICs) of IBG and other antimicrobials were determined by performing the broth microdilution method in accordance with the Clinical and Laboratory Standards Institute. (2018). The MIC is the lowest concentration of IBG observed to inhibit bacterial growth after 24 h of incubation. The minimal bactericidal concentration (MBC) is the lowest concentration that reduces bacterial colonies by 99.9%. The synergistic activity between IBG and antibiotics was measured by using checkerboard assays (MacNair et al., 2018). Fractional inhibitory concentration indices (FICI) were calculated as follows:

FICI=MIC a in combination/ MIC a alone+MIC b in combination/ MIC.b alone

### 2.3 *In vitro* time-killing curves

On the basis of MICs, *R. anatipestifer* ATCC11845 and GDH21D36 were cultured to a concentration of approximately 10^6^ CFU/mL in TSB. Different concentrations (1/4 MIC, 1/2 MIC, 1 MIC, 2 MIC, and 4 MIC) of IBG or GEN (1/4 MIC, 1/2 MIC) were added to the bacterial suspensions, and then inoculated at 37°C with agitation at 180 rpm. A tube of bacterial suspensions without the drug served as the control. All the tubes were incubated at 37°C. At 0, 1, 2, 4, 8, 12, and 24 h, 100 μL of culture was serially diluted, and the solvents were spotted onto a TSA medium. The limit of detection was 10 CFU/mL. Each experiment was performed with three replicates.

### 2.4 Establishment of pericarditis model

Two-week-old Cherry Valley ducks weighting 100 ± 20 g were used in this study. The ducks were provided antibacterial-free balanced feedstuff (CP FEED, Jiangsu) according to labeling and clean water. *R. anatipestifer* ATCC11845 was cultured in TSB and incubated at 37°C for about 16–24 h. Subsequently, bacteria were washed and resuspended in physiological saline to 10^8^ CFU/mL. Pericarditis in the *R. anatipestifer*-infected ducks was induced through the intraperitoneal injection of 0.5 mL of 10^8^ CFU/mL *R anatipestifer* ATCC11845 suspension as described previously (Qiu et al., 2016). Infected ducks received intramuscular injection 4 mg/kg b. w. Of IBG, GEN, and IBG combined with GEN with two times a day for 3 successive days (n = 6). All animal procedures were approved by the Institutional Animal Care and Use Committee of South China Agricultural University (approval number: 2022A007), and the animals were treated with consideration for their welfare and in compliance with all local and national legal requirements.

### 2.5 Serial-passage mutagenesis assay

Overnight cultures of *R. anatipestifer* ATCC11845 were inoculated into TSB containing IBG at 1–8 μg/mL. Bacterial cells were harvested at 24 h after incubation at 37°C. Ciprofloxacin and 1% DMSO were used as a positive and negative control, respectively. Every 24 h, 30% glycerin was added to each tube with bacterial solution. The tubes were then stored at −20°C for serial passage. An MIC assay was performed through the microbroth dilution method. Experiments were performed in triplicates.

### 2.6 Antibacterial activity under exogenous addition

The levels of PE, PG, CA, EDTA, Trixon-X-100, LPS, and different cations (NaCl, CaCl_2_, and MgCl_2_) were analyzed by checkerboard assays to understand the effects of exogenous addition on the antibacterial activity of IBG against *R. anatipestifer* ATCC11845.

### 2.7 Cell membrane integrity assay

Cell membrane integrity assay was performed as a previous report (Song et al., 2020). *R. anatipestifer* ATCC11845 was inoculated into TSB and incubated at 37°C overnight. Bacteria were washed and resuspended in PBS to an OD_600_ of 0.5. Subsequently, the fluorescent probe PI was added at a final concentration of 0.5 μmol/L. A total of 190 μL of the mixture was added to a black 96-well plates after incubation away from light at 37°C for 30 min and added with different concentrations of IBG (final concentrations of 0–16 μg/mL). Bacterial solution (100 μL) was collected from each well and transferred to a black 96-well plate after 30 min of incubation at 37°C. Fluorescence was measured at an excitation wavelength of 535 nm and emission wavelength of 615 nm.

### 2.8 Cell membrane potential assay

The fluorescent probe DiSC_3_(5) was used to determine the effect of IBG on the cell membrane potential (∆Ψ) of *R. anatipestifer* (Hamamoto et al., 2015). Overnight cultures of *R. anatipestifer* ATCC11845 were washed and resuspended in PBS to an OD_600_ of 0.5, and the fluorescent probe DiSC_3_(5) was added at a final concentration of 0.5 μmol/L. After 30 min of incubation at 37°C, 190 μL of the probe-labeled bacterial cells was collected, and 10 μL of IBG (final concentrations of 0–16 μg/mL) was added to a black 96-well plate. The mixture was mixed by blowing and suction and incubated at 37°C for 30 min. The excitation wavelength of the fluorescence spectrometer was 622 nm, and the emission wavelength was 670 nm.

### 2.9 ∆pH assay

Another component of the proton motive force (PMF) is the transmembrane proton gradient (∆pH), which was measured with the pH-sensitive fluorescent probe BCECF-AM (Liu et al., 2020). *R. anatipestifer* ATCC11845 was grown overnight at 37°C. Bacterial cells were washed and suspended in PBS until their OD_600_ normalized to 0.5. A total of 190 μL of BCECF-AM was added at the final concentration of 10 μmol/L to a black 96-well plate and mixed fully with 10 μL of IBG at the final concentrations of 0, 2, 4, 8 and 16 μg/mL. The plate was incubated at 37°C for 30 min and placed in a fluorescence spectrometer with excitation and emission wavelengths of 488 and 535 nm, respectively.

### 2.10 ATP determination

The ATP levels in *R. anatipestifer* ATCC11845 were detected by using an enhanced ATP assay kit (Beyotime, Shanghai, China). Overnight cultured *R. anatipestifer* ATCC11845 cells were washed three times with PBS (pH = 7.4) and resuspended to an OD_600_ of 0.5. The resuspension was added with IBG (final concentrations of 0–16 μg/mL) and incubated at 37°C for 30 min. Subsequently, cultures were centrifuged at 12,000 rpm for 5 min. Supernatants were collected to measure extracellular ATP levels. Pellets were lysed with lysozyme and centrifuged to detect intracellular ATP. ATP levels were measured by using a Hitachi F-7000 fluorescence spectrometer.

### 2.11 Molecular docking

The model structure of the PgsA and PlsB proteins was obtained from the UniProt Knowledgebase (https://www.uniprot.org/uniprotkb accessed on 25 December 2023). The protein sequence was A0A126QFI4 and V4MRX6. The 2D structure of IBG was displayed using ChemDraw 20.0. Molecular docking of PgsA and PlsB proteins with IBG was performed using the LibDock protocol of Discovery Studio 2019.

### 2.12 Data processing

GraphPad Prism 8.0 software was used for statistical analysis. All data were presented as mean ± standard deviation. One-way ANOVA was used to calculate *p* values between multiple groups (ns, not significant, *p < 0.05, **p < 0.01, ***p < 0.001).

## 3 Result

### 3.1 *In vitro* susceptibility testing

The MIC and MBC of IBG against different kinds of bacteria are shown in Table 1. IBG lacked antibacterial activity (MIC >256 μg/mL) against other Gram-negative bacteria. MIC measurements were performed on 30 *R. anatipestifer* isolates with various antibiotic resistance phenotypes to test the antimicrobial activity of IBG (Table 2). IBG showed better *in vitro* antibacterial activity against the clinical isolates than some commonly used antibiotics. The MIC range of IBG for *R. anatipestifer* (n = 50) was 0.5–2 μg/mL. The MIC_50_ and MIC_90_ of IBG were 1 μg/mL. IBG had MBCs of 1–4 μg/mL and the MBC_50_ and MBC_90_ of IBG of 2 μg/mL. The MICs of IBG alone and in combination with antibiotics for *R. anatipestifer* are listed in Table 3. The combination of IBG with GEN showed enhanced activity against *R. anatipestifer* with FICI values that varied from 0.38 to 0.50.

**TABLE 1:** MIC and MBC of IBG for different kinds of bacteria.

Strain	MIC (μg/mL)	MBC (μg/mL)
*Staphylococcus aureus* ATCC 29213	4	16
*Enterococcus faecalis* ATCC 29212	4	8
*Streptococcus suis* ATCC 43765	8	16
*Escherichia coli* ATCC 25922	>256	-
*Salmonella* ATCC 14028	>256	-
*Klebsiella pneumoniae* ATCC 700603	>256	-
*Riemerella anatipestifer* ATCC 11845	2	4

**TABLE 2:** MIC of different antibiotics against *R. anatipestifer* (*n* = 50).

Antibiotics	MIC (μg/mL)
CEQ	0.015–32
CTX	0.03–4
NEO	32–128
GEN	16–64
DOX	1–4
CL	16–64
ENR	2–8
FLR	1–16
STX	32–64
RIF	0.06–32
TMI	8–64
IBG	0.5–2

CEQ, cefquinome; CTX, cefoxitin; NEO, neomycin; GEN, gentamycin; DOX, doxycycline; CL, colistin; ENR, enrofloxacin; FLR, florfenicol; STX, sulfamethoxazole/trimethoprim; RIF, rifamycin; TMI, tilmicoisn; IBG, isopropoxy benzene guanidine.

**TABLE 3:** Antibacterial activity of IBG in combination with antibiotics against *R. anatipestifer*.

	FICI	
	ATCC11845	GDH21D24
IBG + FLR	1	1
IBG + DOX	1	0.75
IBG + ENR	1	0.53
IBG + AMO	0.75	1
IBG + CEF	1	0.75
IBG + GEN	0.50	0.38
IBG + TMI	0.56	0.75
IBG + CL	1.24	1.24
IBG + SMM	2	1

FLR, florfenicol; DOX, doxycycline; ENR, enrofloxacin; AMO, amoxicillin; CEF, ceftiofur; GEN, gentamycin; TMI, tilmicoisn; CL, colistin; SMM, sulfamonomethoxine.

### 3.2 Time-killing assays

**FIGURE 2:**
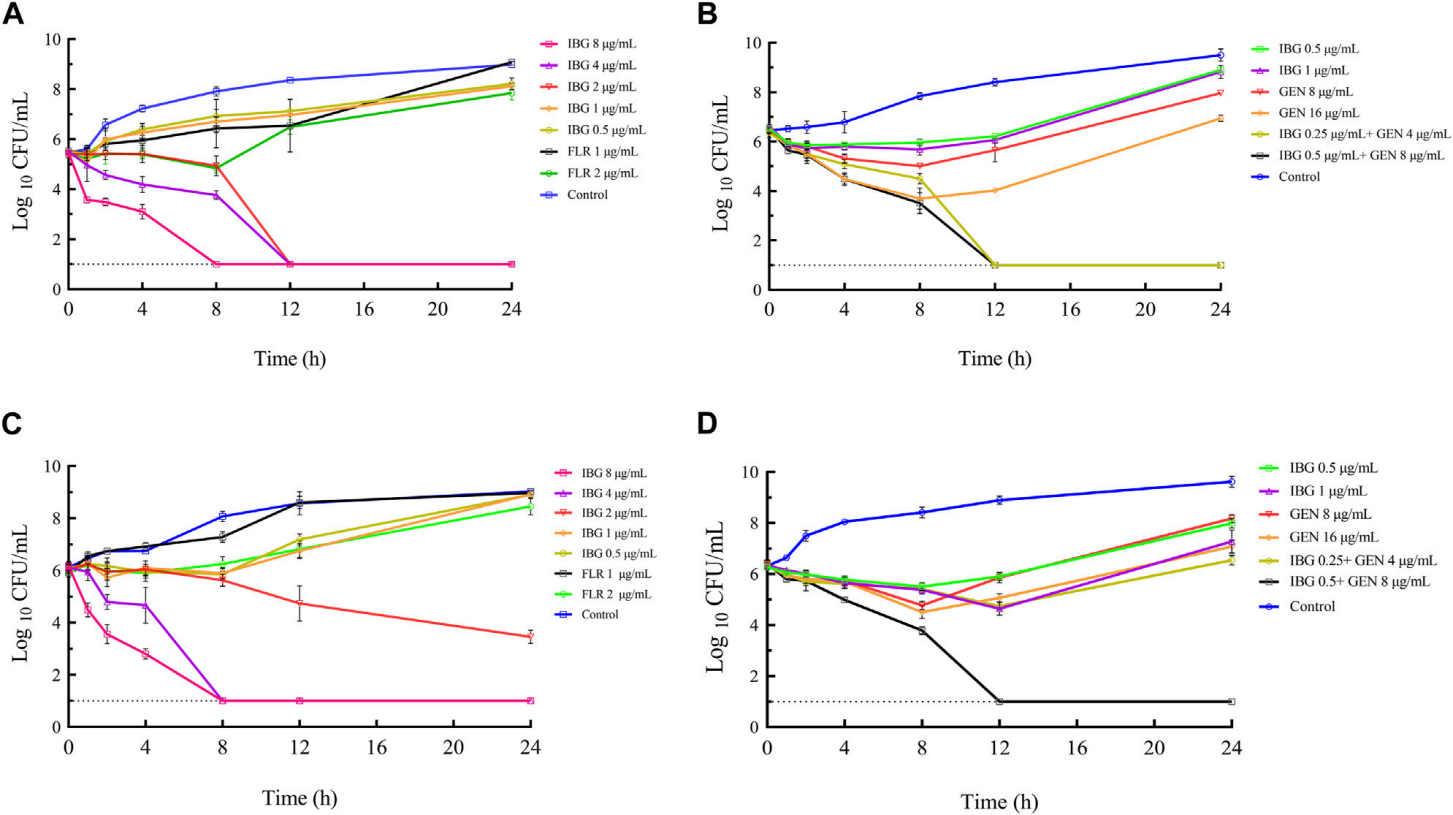
*In vitro* time-killing curves of IBG alone and in combination with GEN against *R. anatipestifer* ATCC11845 **(A, B)** and GDH21D24 **(C, D)**. IBG, isopropoxy benzene guanidine; FLR, florfenicol; GEN, gentamycin.

The time-killing curves of IBG combined with GEN for *R. anatipestifer* ATCC11845 and GDH21D24 in TSB are illustrated in Figure 2. The results showed that antibacterial activity increased with IBG concentration, indicating that the antibacterial effect of IBG on *R. anatipestifer* was concentration-dependent. When the concentration of IBG was less than 1×MIC, the growth of *R. anatipestifer* was slightly inhibited and subsequently resumed (Figures 2A, C). IBG demonstrated bactericidal activity at concentrations exceeding 2 × MIC, with no bacterial regrowth observed within 24 h. Bactericidal effects were observed when IBG and GEN were present at the concentration of 0.25×MIC and less than 1×MIC (Figures 2B, D).

### 3.3 *In vivo* efficacy

**FIGURE 3:**
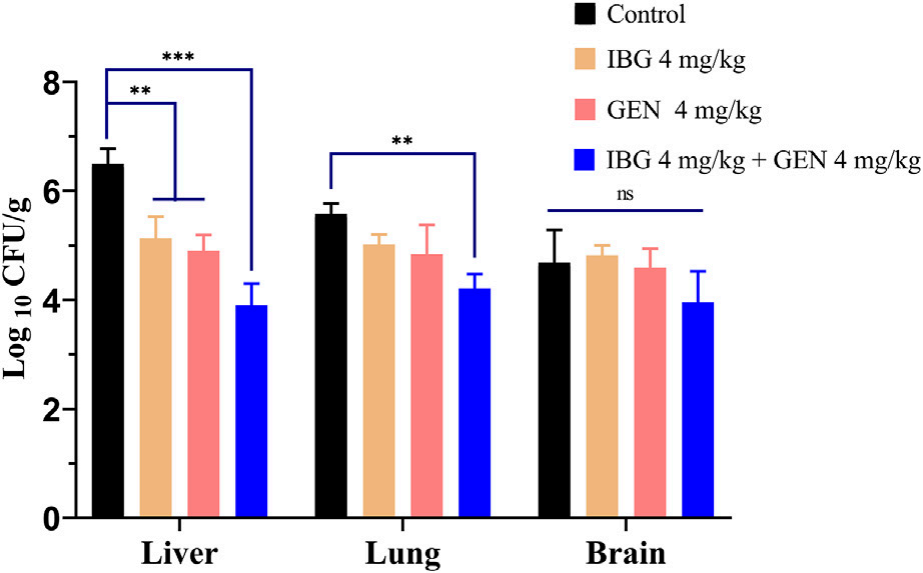
Bacterial loads in the liver, lung, and brain tissues of ATCC11845-infected ducks after treatment with IBG combined with GEN. (ns, not significant, *p < 0.05, **p < 0.01, ***p < 0.001.)

The bacterial burden in lung, liver, and brain tissues of infected ducks without drug treatment was 5.59 ± 0.74 log_10_ CFU/g. The bacterial burden in the liver of ducks treated with IBG and GEN significantly reduced (*p* < 0.01) compared with that in the untreated control (Figure 3). The injection of 4 mg/kg GEN with 4 mg/kg IBG significantly increased the antibacterial activity in the lung (*p* < 0.01) and liver (*p* < 0.001), reducing the bacterial load to 1.37–2.60 log_10_ CFU/g.

### 3.4 Serial-passage mutagenicity assay

**FIGURE 4:**
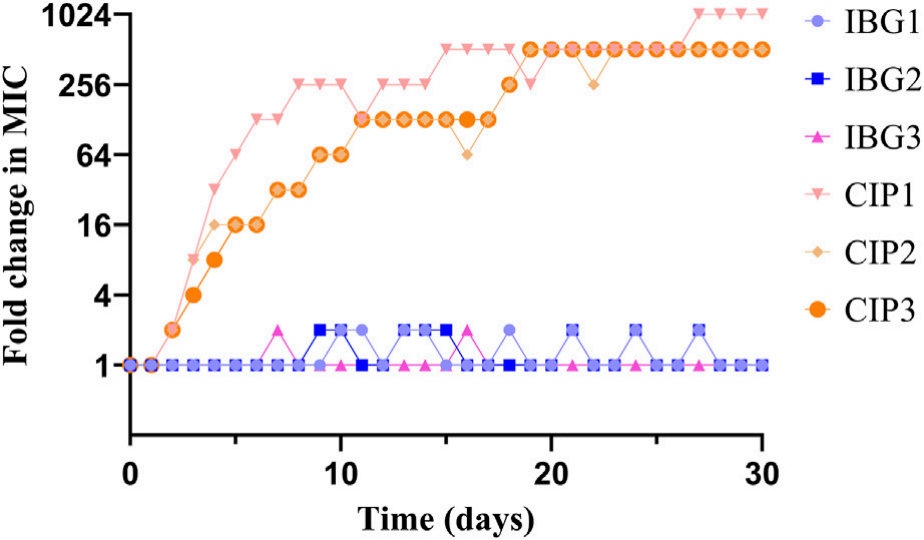
Changes in the MICs of IBG and CIP for *R. anatipestifer* ATCC 11845 after 30 days of serial passage.

In resistance studies, *R. anatipestifer* ATCC11845 was continuously passaged under the subinhibitory concentration of IBG. Under the pressure of IBG, the MIC of IBG for *R. anatipestifer* only increased two times within 30 days (Figure 4). By contrast, the MIC of CIP increased 256 times within 30 days.

### 3.5 IBG disrupted the *R. anatipestifer* cell membrane

**FIGURE 5:**
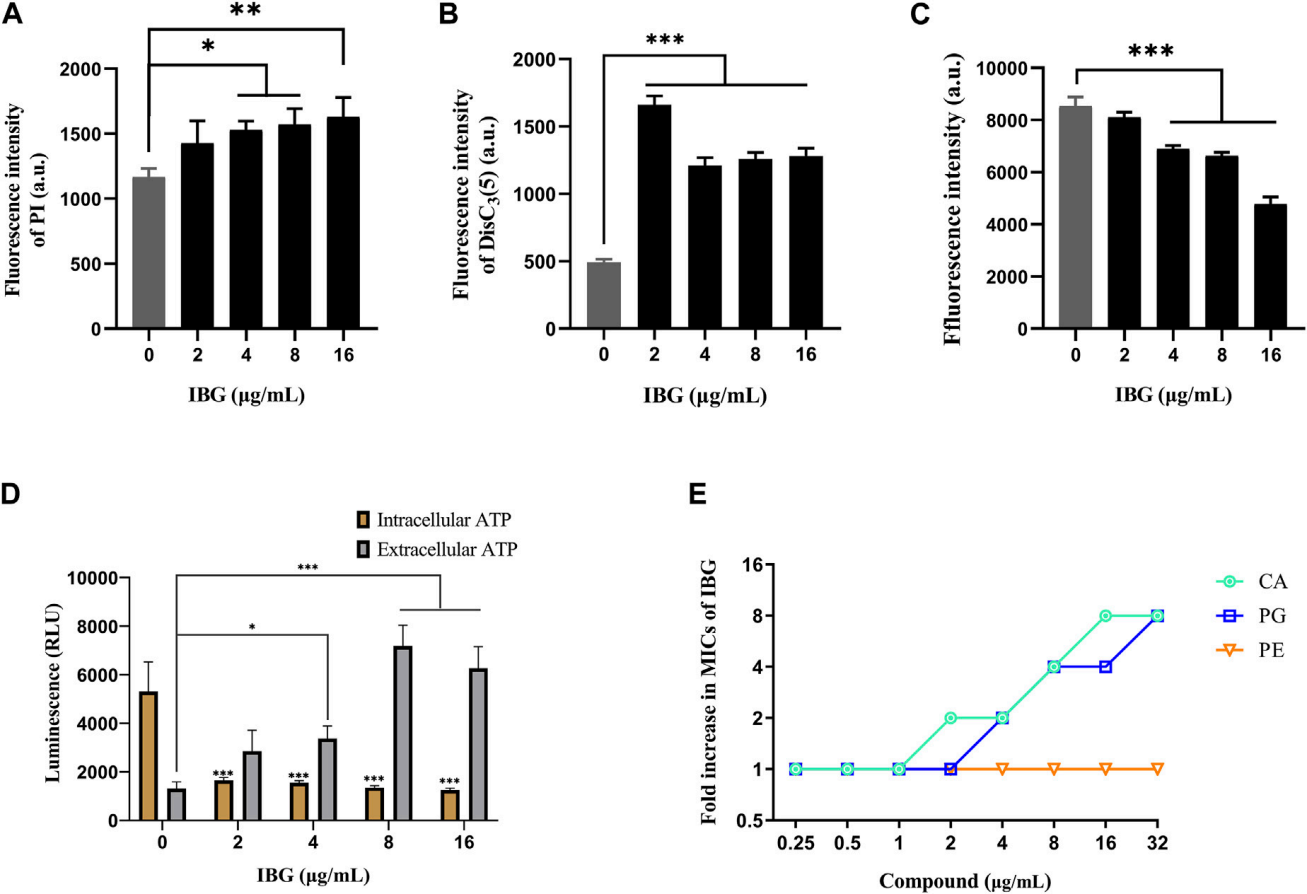
Mechanism of IBG against *R. anatipestifer*. **(A)** Increased permeability of the inner membrane of *R. anatipestifer* ATCC11845 treated with different concentrations of IBG. **(B)** The fluorescent probe DiSC_3_(5) was used to detect the membrane potential. **(C)** ΔpH of *R. anatipestifer* ATCC11845 treated with IBG was obtained by using BCECF-AM probes **(D)** Intracellular and extracellular ATP levels in *R. anatipestifer* ATCC11845 treated with different concentrations of IBG. **(E)** Antibacterial activity of IBG combined with CA, PG, or PE. All data were expressed as mean ± standard deviation, and significance was determined by nonparametric one-way ANOVA. (ns, not significant, *p < 0.05, **p < 0.01, ***p < 0.001.)

*R. anatipestifer* ATCC 11845 was used as an indicator to explore the anti-*R. anatipestifer* mechanism of IBG. The fluorescence probe PI was used to measure the cell membrane integrity of *R. anatipestifer* after IBG treatment (Song et al., 2020). The results showed that IBG increased fluorescence intensity in a concentration-dependent manner (Figure 5A). A significant difference (*p* < 0.05) was found between the IBG-treated and control groups. These results indicated that in *R. anatipestifer*, IBG can disrupt the integrity of the cell membrane and induce membrane damage and cytoplasmic membrane dysfunction.

**FIGURE 6:**
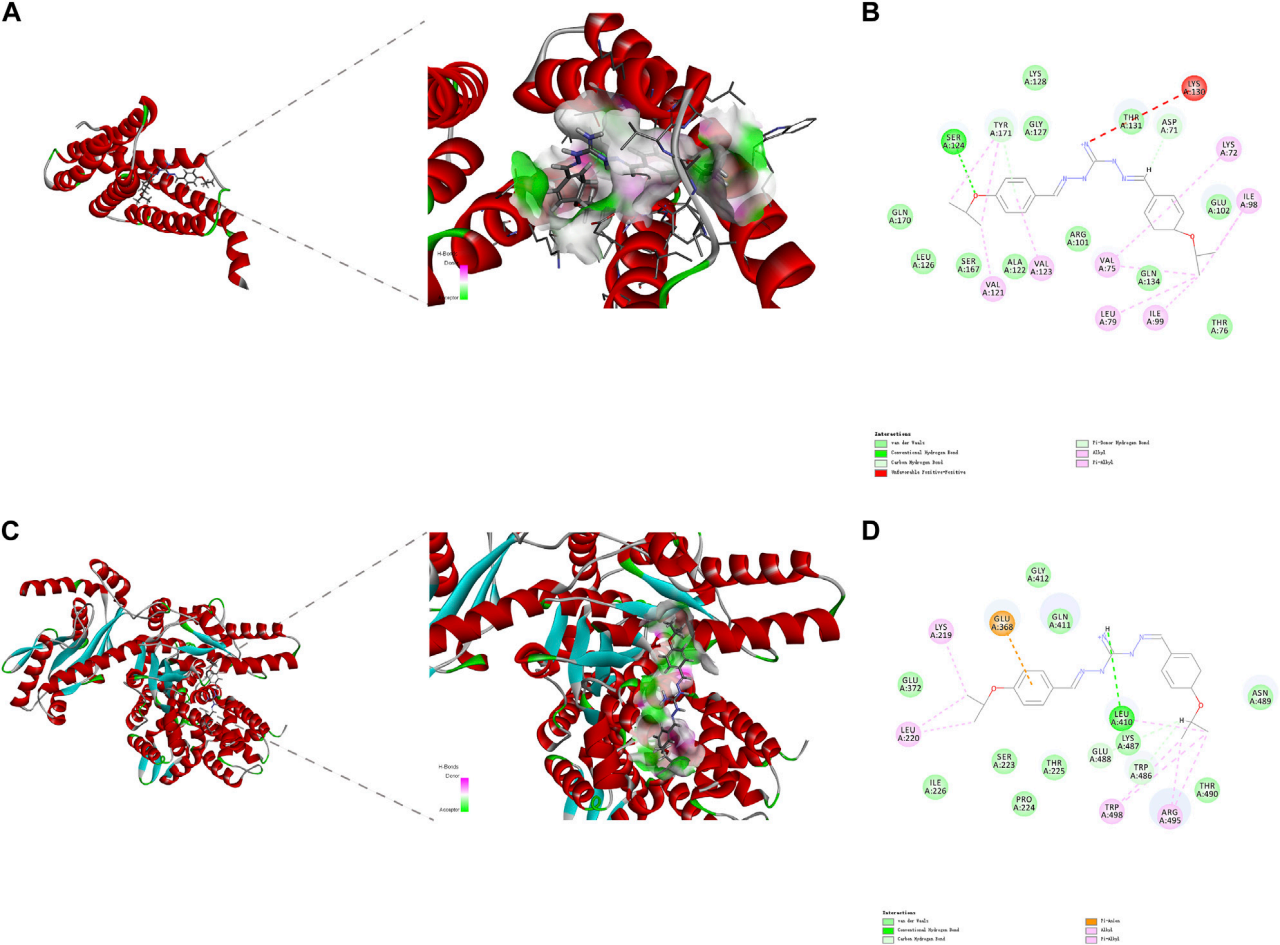
The interaction pattern between IBG and the proteins PgsA **(A, B)** and PlsB **(C, D)**.

DiSC_3_(5) was used to determine changes in membrane potential in *R. anatipestifer* after IBG treatment (Hamamoto et al., 2015). The fluorescence in the experimental group significantly increased (*p* < 0.001), and IBG significantly increased the membrane potential of *R. anatipestifer* (Figure 5B). Given that IBG can affect ΔΨ, BCECF-AM was used to evaluate the effect of IBG on the *Δ* pH of *R. anatipestifer*. Compared with that of the control group, the membrane potential of the IBG group had significantly reduced (*p* < 0.001) in a concentration-dependent manner (Figure 5C). Given that PMF disruption affects cellular ATP (Vahidi et al., 2016), intracellular and extracellular ATP levels were measured. IBG decreased intracellular ATP levels and increased extracellular ATP levels (Figure 5D). Next, investigated the effect of major cytoplasmic membrane components on the activity of IBG against *R. anatipestifer* ATCC 11845 under exogenous addition was investigated. The exogenous addition of bacterial phospholipids (including PG and CA) inhibited IBG activity in a dose-dependent manner (Figure 5E). The proteins PgsA and PlsB play a crucial role in the synthesis of PG and CA (Li et al., 2016). To investigate the binding interactions between IBG and these proteins, molecular docking was conducted. The results demonstrated a favorable affinity between IBG and PgsA and PlsB, as indicated by LibDockScores of 104.70 and 77.65, respectively. Additionally, the molecular docking analysis revealed potential interactions between IBG and the proteins PgsA and PlsB. In the case of the PgsA protein, the binding sites of IBG were found to contain potentially critical active residues, including TYR171, SER124, VAL121, VAL123, LYS130, ASP71, VAL75, LYS72, LEU79, ILE99, and ILE98 (Figures 6A, B). For the PlsB protein, potentially critical active residues include LEU220, LYS219, GLU368, LEU410, LYS487, GLU488, TRP486, and ARG495 (Figures 6C, D).

## 4 Discussion

Given that antibiotic resistance is becoming an increasingly serious problem, finding novel antibacterial drugs is a means for effectively controlling infections by drug-resistant bacteria (De Oliveira et al., 2020; Huemer et al., 2020). Guanidine compounds are used to treat various diseases and are candidates for the structural modification of novel drugs (Kim et al., 2021). Metformin is a commonly prescribed medication for managing diabetes (Foretz et al., 2023). When combined with tetracyclines, it has a good synergistic antibacterial effect on methicillin-resistant *S. aureus* (Liu et al., 2020). The guanidine compound H-BDF has a good antibacterial against *Pseudomonas aeruginosa* and a synergistic antibacterial effect with meropenem or ciprofloxacin (Saeed et al., 2018). Guanidine compounds, especially substituted phenylguanidine derivatives, possess a long history and promising application prospects (Kelly et al., 2015; Previtali et al., 2020). Robenidine was initially employed during the early 1970s for the treatment of coccidiosis in poultry and rabbits (Holdsworth et al., 2004). Additionally, it exhibits antibacterial activity against *Candida albicans* (Sorribas et al., 1993; Mei et al., 2020). Some researchers modified the structure of robenidine and obtained the analog NCL195, which has antibacterial activity against *Streptococcus pneumoniae* and *S. aureus* (Pi et al., 2020). Several chlorobenzene guanidine analogs were obtained through the structural modification of chlorobenzene guanidine, which has antibacterial activity against vancomycin-resistant *Enterococcus*, methicillin-resistant *S. aureus*, and *E. coli* (Abraham et al., 2016). In the present study, we found that substituted phenylguanidine derivatives showed excellent antibacterial activity against *R. anatipestifer* (MIC ≤2 μg/mL) and concentration-dependent antibacterial activity.

The emergence and rapid dissemination of antibiotic resistance among bacteria pose a significant threat to the health of both humans and animals (Watkins and Bonomo, 2016). Studying the development of drug resistance in bacteria under laboratory conditions is convenient and inexpensive. *R. anatipestifer was* passaged serially under IBG pressure. The MIC of IBG for IBG-resistant strains showed a low likelihood of increasing within 30 days, with only an increase two times in certain passage days. Within a span of 14 days, the MIC in the CIP group exhibited an increase from 0.03 to 4 μg/mL. Following a 20 days exposure to sub-inhibitory concentration of rifampicin, the MIC of *S. aureus* ATCC 25923 was increased rapidly from 0.032 to 256 μg/mL (Zhang et al., 2023). This result indicated that *R. anatipestifer* does not easily acquire resistance to IBG. Furthermore, cross-resistance between IBG and conventional antibiotics was not observed.

**FIGURE 7:**
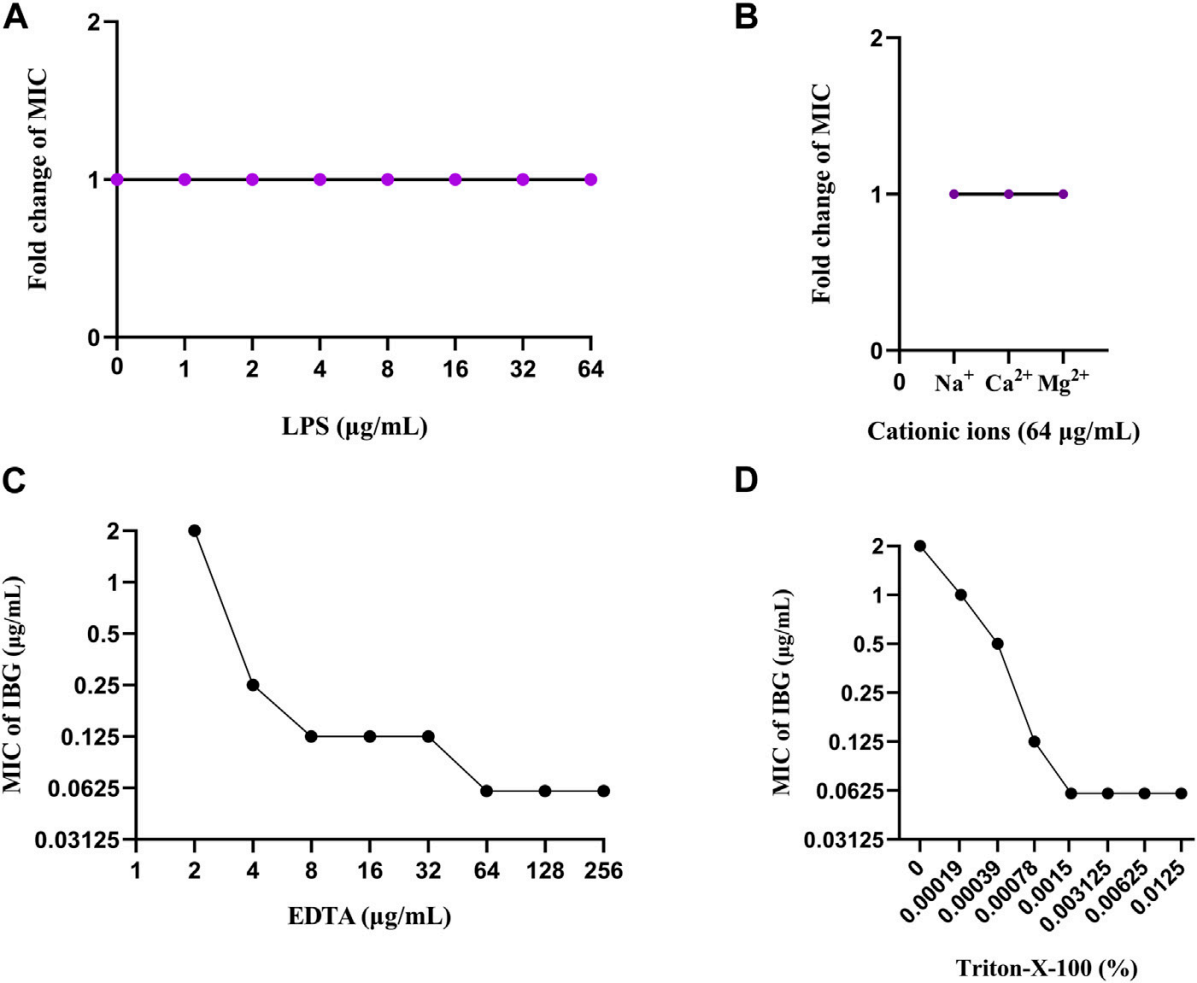
Change in the MICs of IBG for *R. anatipestifer* ATCC11845 in the presence of 0–64 μg/mL LPS **(A)**. Changes in the MICs of IBG for *R. anatipestifer* ATCC11845 in the presence of different cations at a concentration of 64 μg/mL **(B)**. Change in the MIC of IBG against *R. anatipestifer* ATCC11845. Synergy of IBG with EDTA **(C)** and Triton-X-100 **(D)** against *R. anatipestifer* ATCC11845 was explored through checkerboard microdilution.

Notably, IBG lacks antibacterial activity against Gram-negative bacteria, except *R. anatipestifer*. Given that the phospholipid compositions of the cell membranes of Gram-positive and negative bacteria are the same (Dias et al., 2018), it can be speculated that the outer membrane of Gram-negative bacteria (except *R. anatipestifer*) prevents IBG from reaching phospholipids. The impact of exogenous LPS and divalent cations on the activity of IBG was to eliminate the potential influence of the outer membrane (Bonnington and Kuehn, 2016; MacNair and Brown, 2020). Exogenous LPS and divalent cations had negligible effects on IBG activity (Figures 7A, B). Furthermore, the membrane penetrants EDTA and Triton-X-100 enhanced the activity of IBG against *R. anatipestifer*. (Figures 7C, D). This effect was consistent with that of IBG on *S. aureus* and *E. coli*, suggesting that the outer membrane provides a physical barrier. In Gram-negative bacteria, the specific permeability of the outer membrane is the main component that hinders the entry of most drugs (Sperandeo et al., 2017). IBG has completely different antibacterial effects on Gram-positive and negative bacteria, and even its antibacterial effects on different Gram-negative bacteria are not exactly the same. Thus, we speculated that differences in outer membrane structures is the main reason why IBG has antibacterial activity against *R. anatipestifer* but not against other Gram-negative bacteria.

We used PI to detect the effect of IBG on the integrity of the *R. anatipestifer* membrane to explore the anti-*R. anatipestifer* mechanism of IBG (Song et al., 2020). Consistent with the effect of IBG on *S. aureus*, IBG increased fluorescence intensity in a concentration-dependent manner, (Li et al., 2022). Bacterial PMF is an energy pathway located on the cell membrane of a bacterium and executes an important regulatory role in the synthesis of ATP, active transport of molecules, and rotation of bacterial flagellum (Yang et al., 2023). The PMF of bacteria binds sites and can be used to develop antibacterial agents and synergists (Hubbard et al., 2017; Stokes et al., 2020; Liu et al., 2021; Tong et al., 2021). In the present study, DiSC_3_(5) and BCECF-AM were employed to observe alterations in ΔΨ and ΔpH, which are generally encompassed within the PMF (Chen and Lo, 2016; Liu et al., 2020). Following the administration of IBG to *R. anatipestifer*, the dissipation of ΔΨ and ΔpH was observed. Therefore, IBG can play an antibacterial role against *R. anatipestifer* by interacting with PMF. IBG mainly exerts its antibacterial effect by binding to the cytoplasmic membrane. After the exogenous addition of PG and CA, the main cytoplasmic membrane components effectively inhibited the antibacterial activity of IBG, providing evidence supporting the action of IBG as a PG- and CA-targeting antibiotic.

Based on the above results, IBG exhibits promise as a potential compound for addressing *R. anatipestifer* infections. However, the utilization of guanidine compounds in animals may be hindered by challenges such as limited solubility, inadequate bioavailability, and side effects (Kawabata et al., 2011; Kalepu and Nekkanti, 2015). Consequently, future endeavors in the development and application of IBG should prioritize the identification of an appropriate dosage form and a rational dosage regimen to mitigate any potential toxicological repercussions.

## 5 Conclusion

The antibacterial activity of IBG against *R. anatipestifer* may be due to the great difference between the outer membrane components of *R. anatipestifer* and those of other Gram-negative bacteria, such as *E. coli*. Thus, IBG can permeate the outer membrane successfully. IBG triggers cytoplasmic membrane damage by binding to PG and CA, leading to the dissipation of PMF and reductions in intracellular ATP. IBG is a potential compound for the treatment of *R. anatipestifer* infections.

## Data Availability

The original contributions presented in the study are included in the article/Supplementary Material, further inquiries can be directed to the corresponding author.
